# Acute Hemoperitoneum Secondary to Chronic Hematosalpinx: A Diagnostic Challenge in the Setting of a Negative Pregnancy Test

**DOI:** 10.7759/cureus.97333

**Published:** 2025-11-20

**Authors:** Liane Silva, Abdelaziz Satti, Hani Bashir, Alison DeMaio

**Affiliations:** 1 Obstetrics and Gynaecology, Tallaght University Hospital, Dublin, IRL; 2 Obstetrics and Gynaecology, Coombe Hospital, Dublin, IRL

**Keywords:** acute hemoperitoneum, chronic haematosalpinx, emergency laparoscopy, endometriosis, rare cause of acute abdominal pain

## Abstract

Acute hemoperitoneum represents a gynecological and surgical emergency with significant morbidity if diagnosis and intervention are delayed. In women of reproductive age, the etiology is most frequently ruptured ectopic pregnancy; however, when pregnancy testing is negative, the differential diagnosis becomes considerably broader and less well defined.

Hematosalpinx, although most commonly associated with ectopic gestations, may also occur secondary to non-pregnancy-related etiologies, including endometriosis, pelvic inflammatory disease, or primary tubal pathology. In rare instances, chronic hematosalpinx can rupture or leak, leading to substantial intraperitoneal hemorrhage and clinical features that mimic more common acute abdominal emergencies. This diagnostic uncertainty may obscure timely recognition and delay appropriate management.

This case demonstrates acute hemoperitoneum secondary to chronic hematosalpinx in the context of a negative pregnancy test and highlights the diagnostic challenge. Pelvic and transvaginal ultrasound usually represents the first-line imaging modality for suspected adnexal pathology, aiding differentiation between hematosalpinx and ruptured ectopic pregnancy before proceeding to CT or surgical exploration. Early recognition and prompt surgical intervention are essential for accurate diagnosis, definitive treatment, and preservation of reproductive potential.

## Introduction

Background

Hematosalpinx is a medical condition involving bleeding into the fallopian tubes [1,2]. The most common cause of a hematosalpinx is ectopic pregnancy, though other causes include pelvic inflammatory disease (PID), endometriosis, and pelvic trauma [3]. A hematosalpinx refers to intraluminal blood within the fallopian tube (often dilated) and may present as dilated fallopian tubes with homogeneous low-level echoes on imaging [4,5].

Ectopic pregnancy becomes chronic when there are multiple small hemorrhages into the peritoneal cavity with the formation of a pelvic hematocele. Chronic ectopic pregnancy has relatively less acute symptoms and rarely presents as a large hematosalpinx without classical symptoms [2].

Chronic hematosalpinx may have multiple underlying causes and is associated with gynecological disorders. Individuals with female reproductive anatomy, especially those who are of reproductive age, are predominantly affected by hematosalpinx [6]. The various etiologies include tubal ectopic pregnancy (the most common cause); endometriosis affecting the fallopian tubes, where cyclical hemorrhage of endometrial implants can cause distension of the fallopian tubes, leading to hematosalpinx formation [7]; tubal carcinoma; PID; and fallopian tube torsion.

The mechanism of tubal pathology leading to hematosalpinx involves mechanical blockage of adnexal veins and lymphatic vessels, leading to local edema and pelvic congestion. If there is an associated compromise in the arterial supply, it leads to hemorrhagic infarction and, subsequently, a hematosalpinx [8]. Isolated torsion of the fallopian tube is an uncommon cause of acute lower abdominal pain, with an incidence estimated to be 1 in 500,000 women [9].

The clinical presentation can vary significantly. Symptoms may include abdominal or pelvic pain, which may be acute or chronic, and abnormal vaginal bleeding [6]. A hematosalpinx from a tubal pregnancy may be associated with pelvic pain and uterine bleeding, while a hematosalpinx from other conditions may be painless but could lead to uterine bleeding [3]. Blood may also escape into the peritoneal cavity, leading to hemoperitoneum, which can present as an acute surgical emergency.

## Case presentation

A 41-year-old woman, para 2, presented to the emergency department of an acute general hospital with an acute onset of severe lower abdominal pain that began on Saturday night and significantly worsened on Sunday morning. She described the pain as 10/10 intensity, sharp in nature, and accompanied by lightheadedness, sweating, and nausea. She reported minimal vaginal bleeding since the previous day and denied urinary symptoms, bowel symptoms, or vomiting.

Her medical history was significant for an intrauterine contraceptive device (Mirena) in situ for the past five years with resultant amenorrhea, a previous ectopic pregnancy managed conservatively, and no history of ovarian cysts or previous surgical procedures. She had no known drug allergies.

On physical examination, her vital signs initially demonstrated relative hemodynamic stability with a blood pressure of 115/78 mmHg, pulse of 96 bpm, temperature of 36.5°C, respiratory rate of 18 breaths per minute, and oxygen saturation of 100%. Her early warning score was 1. While she appeared unwell, she remained hemodynamically stable throughout the initial assessment. Cardiovascular examination revealed regular pulse rhythm with normal heart sounds and no peripheral edema. Abdominal examination showed a soft abdomen that was tender with mild distension.

Following initial assessment and investigations at the acute general hospital, computed tomography imaging revealed moderate hemoperitoneum with suspicion of an ectopic pregnancy or ruptured hemorrhagic cyst. Given these findings and the patient's clinical presentation, our tertiary gynecological unit (Tallaght University Hospital) was contacted for possible transfer. The patient was subsequently transferred, specifically due to a dropping hemoglobin level, which raised concerns about ongoing intra-abdominal bleeding.

Upon arrival at our tertiary unit, all initial examinations were confirmed, and a comprehensive gynecological examination was performed for the first time. This revealed normal external genitalia with no active bleeding visible, a healthy-appearing cervix with visible Mirena strings in situ, and no abnormal discharge. The patient's clinical condition and laboratory parameters were closely monitored, with serial hemoglobin measurements demonstrating a continuing downward trend.

Investigations

Initial laboratory investigations at the acute general hospital revealed a negative urine pregnancy test and undetectable serum β-hCG levels (<6 mIU/mL). The patient's initial hemoglobin was 12 g/dL. Computed tomography of the abdomen and pelvis with intravenous contrast demonstrated moderate hemoperitoneum with particular concentration in the pouch of Douglas. A serpiginous ill-defined contrast blush was identified on the left side, with free contrast noted in the left paracolic gutter. These imaging findings were highly suspicious for acute gynecological bleeding. The appearances were consistent with blood products within a distended fallopian tube, which typically manifest as high attenuation on CT imaging. On T1-weighted fat-suppressed sequences, the distended tube would be expected to show high signal intensity due to the presence of blood products. Importantly, the CT scan excluded acute appendicitis, mechanical bowel obstruction, and bowel perforation.

Following transfer to the tertiary center, serial hemoglobin measurements revealed a progressive decline from the initial value of 12 g/dL to 9.8 g/dL throughout monitoring. The white cell count was elevated at 15,000/μL, while coagulation studies, including prothrombin time and activated partial thromboplastin time, remained within normal parameters.

Incidental radiological findings included a non-obstructive 7mm calyceal stone on the left kidney, a small hiatus hernia, and simple hepatic and renal cysts, none of which were considered clinically significant in the acute setting.

The combination of negative pregnancy markers, progressive anemia in the context of demonstrated hemoperitoneum, and CT evidence of contrast extravasation supported the decision for urgent surgical intervention.

Differential diagnosis

The acute presentation of severe abdominal pain with hemoperitoneum in a woman of reproductive age required consideration of several important differential diagnoses. Ruptured ectopic pregnancy was the primary concern given the patient's history of previous ectopic pregnancy, acute severe abdominal pain, and imaging evidence of hemoperitoneum. However, the negative urine and serum β-hCG tests made this diagnosis less likely, though the presence of an intrauterine contraceptive device increased the relative risk of ectopic pregnancy should conception occur.

Ruptured hemorrhagic ovarian cyst could also present as an acute presentation and hemoperitoneum, though the patient's five-year history of amenorrhea due to the Mirena device made ovulation and subsequent ovarian cyst formation less likely. Tubal torsion with hematosalpinx became more plausible as the case evolved, particularly given the eventual surgical findings, as isolated torsion of the fallopian tube can lead to hemorrhagic infarction and hematosalpinx formation.

Endometriosis-related bleeding affecting the fallopian tubes was considered as a viable etiology, particularly in a woman with negative β-hCG, as cyclical hemorrhage of endometrial implants can cause distension of the fallopian tubes and hematosalpinx formation.

The combination of negative pregnancy tests significantly narrowed the differential diagnosis, imaging findings of hemoperitoneum with contrast extravasation, progressive decline in hemoglobin, and ultimately the laparoscopic findings of hematosalpinx without evidence of ectopic pregnancy.

Treatment

**Figure 1:**
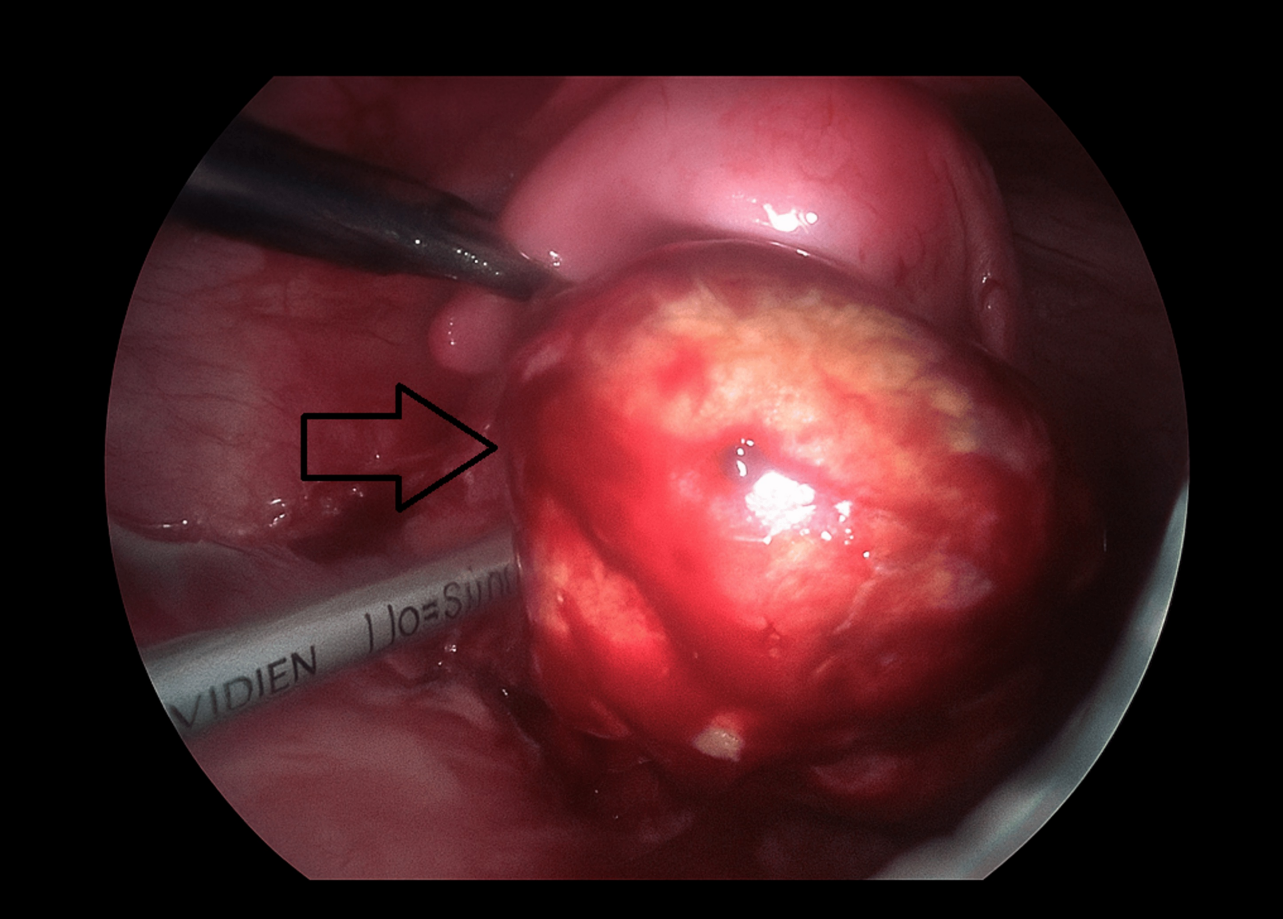
Intraoperative and laparoscopic view showing left adnexa with hematosalpinx (arrow). This intraoperative image demonstrates a left adnexa, with a visibly dilated hematosalpinx showing hemorrhagic discoloration (arrow).

**Figure 2:**
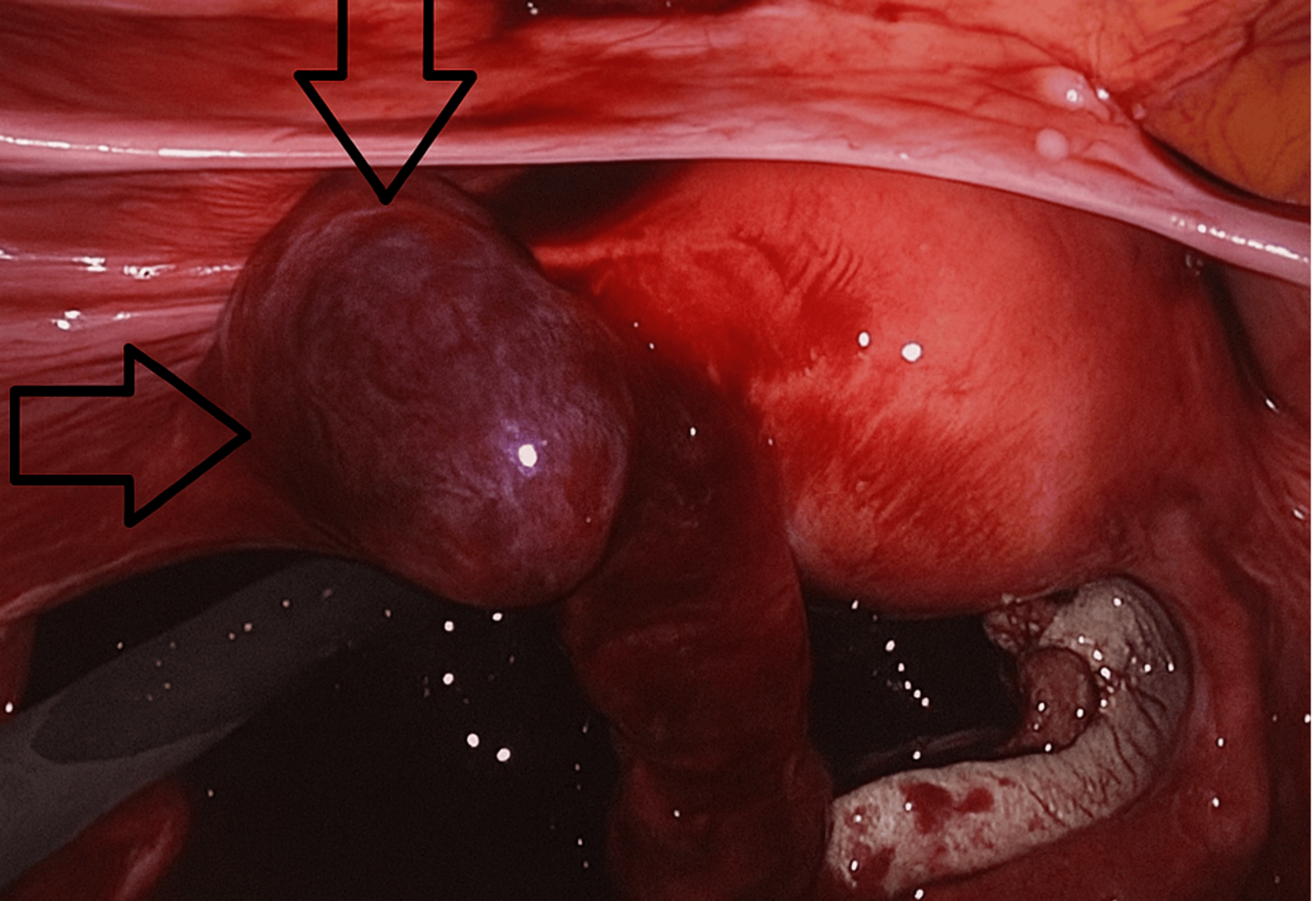
Intraoperative image demonstrating the dilated haematosalpinx with areas of hemorrhagic discoloration.

**Figure 3:**
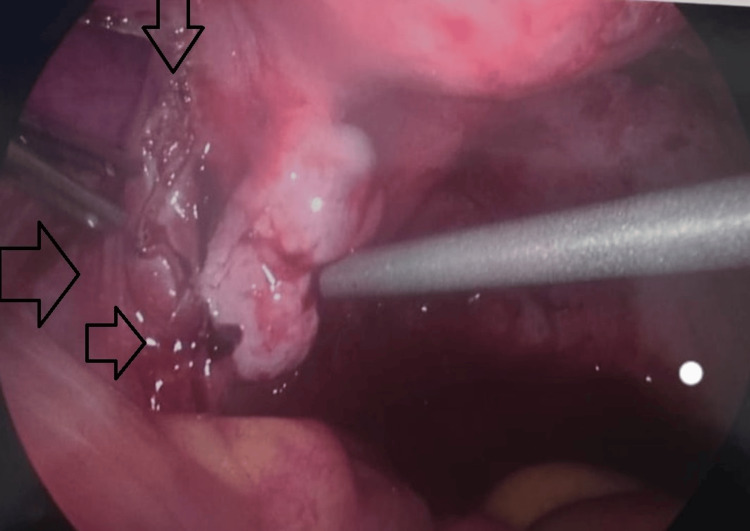
Laparoscopic image demonstrating the left fallopian tube distended with blood (hematosalpinx) and evidence of intraluminal clot. Surrounding hemoperitoneum is also visible.

Based on the clinical presentation and image findings demonstrating hemoperitoneum with progressive decline in hemoglobin levels, the patient was managed with intravenous fluid resuscitation, appropriate analgesia, and nil per oral status in preparation for emergency surgical intervention. Emergency laparoscopic surgery was performed, revealing hematosalpinx (approximately 800 mL of hemoperitoneum). A laparoscopic left salpingectomy was then performed to address the bleeding and remove the affected tissue, and a normal contralateral (right) fallopian tube and ovary were confirmed, with no evidence of active bleeding, adhesions, or endometriotic deposits. The minimally invasive approach allowed for both definitive diagnosis and therapeutic intervention while minimizing surgical morbidity (Figures 1-3). The patient experienced an uncomplicated post-operative recovery, remained hemodynamically stable throughout, and was discharged home in stable condition on the following day with appropriate follow-up arrangements.

Outcome and follow-up

The pathology report from the laparoscopic salpingectomy revealed multiple fragments of tan tissue consisting of hemorrhage, fallopian tube, and grey fibrinous material, with tissue measuring 95 x 70mm in aggregate dimension. No obvious fetal parts were identified, and the fimbrial end was not readily identifiable. The features were consistent with hematosalpinx, though the exact cause remained uncertain from the sample. Importantly, no chorionic villi or specific histological features of ectopic pregnancy were identified, and no evidence of malignancy was detected.

The patient experienced an uncomplicated post-operative recovery with careful monitoring of vital signs and drain output. She remained hemodynamically stable throughout the post-operative period and was discharged home in stable condition on the following day. A six-week follow-up was arranged to review and assess recovery, discuss histopathology results, and counsel regarding future fertility and contraceptive options.

## Discussion

This case report presents a rare presentation of chronic hematosalpinx that presented as an acute surgical emergency with hemoperitoneum. Hematosalpinx is the accumulation of blood in the fallopian tubes, and ectopic pregnancy is the most common cause. Other causes include PID, endometriosis, and pelvic trauma [4]. In this case, with negative pregnancy tests and the absence of chorionic villi on histological examination, the hematosalpinx appeared to be of non-pregnancy-related etiology.

It is rare for a chronic ectopic pregnancy to present as a hematosalpinx. Chronic ectopic pregnancy typically results from small but recurrent bleeding in the pelvic peritoneal cavity. Under certain conditions, this bleeding accumulates within the tube itself to form a hematosalpinx that does not communicate with the peritoneal cavity [2].

The symptoms and signs of chronic ectopic pregnancy are not classical. Adding to the confusion are the negative results of relevant investigations, including negative urine analysis for pregnancy and negative intra-abdominal aspiration [2]. The exact mechanism in this patient remains unclear; however, several possibilities exist. Given the patient's history of previous ectopic pregnancy managed conservatively and current intrauterine contraceptive device (IUCD) placement for five years, chronic inflammatory changes or endometriosis affecting the fallopian tubes could have led to cyclical hemorrhage and hematosalpinx formation [7].

The mechanism involves the mechanical blockage of the adnexal veins and lymphatic vessels, leading to local edema and pelvic congestion, which subsequently compromises the arterial supply, resulting in hemorrhagic infarction and hematosalpinx [8]. The acute presentation of hemoperitoneum and progressive decline in hemoglobin levels suggest rupture or leakage of the blood-filled fallopian tube, creating a surgical emergency requiring urgent transfer to a tertiary center.

The diagnostic challenge in this case was significant, highlighting the importance of inter-hospital collaboration. MRI can be used in cases of diagnostic difficulties. Structural pathology can be studied, and the surgical procedure can be planned with more clarity. The presence of blood (hematosalpinx) within the tube often results in a high signal intensity on T1-weighted images, which is best demonstrated by fat suppression [2]. Clinicians should consider endometriosis as a potential cause in women with negative β-hCG levels [5]. CT findings of hemoperitoneum with contrast extravasation at an acute general hospital were crucial for prompt immediate referral to a tertiary center.

The presence of a gestational sac with or without an embryo in the hematosalpinx (seen as an enhancing thick-walled ring-like structure) is a conclusive sign of ectopic pregnancy within the fallopian tube. However, the most common appearance of a tubal ectopic is a heterogeneous adnexal mass, with or without the ipsilateral ovary seen separately [2].

Treatment is customized based on etiology, degree of severity of symptoms, and reproductive objectives [6]. Surgical management was indicated for this acute presentation of hemodynamic compromise and hemoperitoneum. Every effort is made to protect the fallopian tubes when a patient wishes to conceive. However, if the fallopian tube does not function, in vitro fertilization (IVF) or other reproductive procedures may be advised [6].

The laparoscopic approach was appropriate and allowed for both definitive diagnosis and treatment while minimizing surgical morbidity. With increasing awareness and training in laparoscopy, conservative surgeries such as salpingectomy can be performed laparoscopically, even in rural areas [2]. The successful outcome demonstrated the effectiveness of minimally invasive techniques in managing acute gynecological emergencies. This highlights the importance of establishing appropriate referral pathways between acute general hospitals and tertiary centers.

## Conclusions

This case highlights hematosalpinx as an important differential diagnosis for acute abdominal pain in women of reproductive age, particularly when pregnancy tests are negative and imaging demonstrates hemoperitoneum. The progressive decline in hemoglobin levels in conjunction with CT evidence of intra-abdominal bleeding necessitated urgent transfer to a tertiary gynecological center for specialized intervention.

Histopathological examination of the resected fallopian tube confirmed the presence of hemorrhage and fibrinous material consistent with hematosalpinx, while notably excluding the presence of chorionic villi or fetal tissue that would indicate ectopic pregnancy, and ruling out malignancy. In patients presenting with negative β-hCG levels, pelvic pain, and imaging suggestive of adnexal pathology, endometriosis should be considered as a potential underlying cause of hematosalpinx formation, particularly when histological examination fails to identify pregnancy-related tissue.

Chronic hematosalpinx remains a diagnostic challenge for clinicians due to its atypical presentation and can manifest as hematosalpinx, requiring advanced imaging modalities such as MRI and definitive laparoscopic evaluation for accurate diagnosis. Early recognition, prompt diagnosis, and timely surgical intervention are essential for optimal symptom management, prevention of complications, and preservation of reproductive potential, emphasizing the critical importance of established referral pathways and effective communication between acute general hospitals and tertiary gynecological centers in managing complex gynecological emergencies.
